# Supplementary figures and images for: Boldine Alters Serum Lipidomic Signatures after Acute Spinal Cord Transection in Male Mice
Source: Int J Environ Res Public Health. 2023 Aug 17;20(16):6591. doi: 10.3390/ijerph20166591 (PMC10454893; doi:10.3390/ijerph20166591)

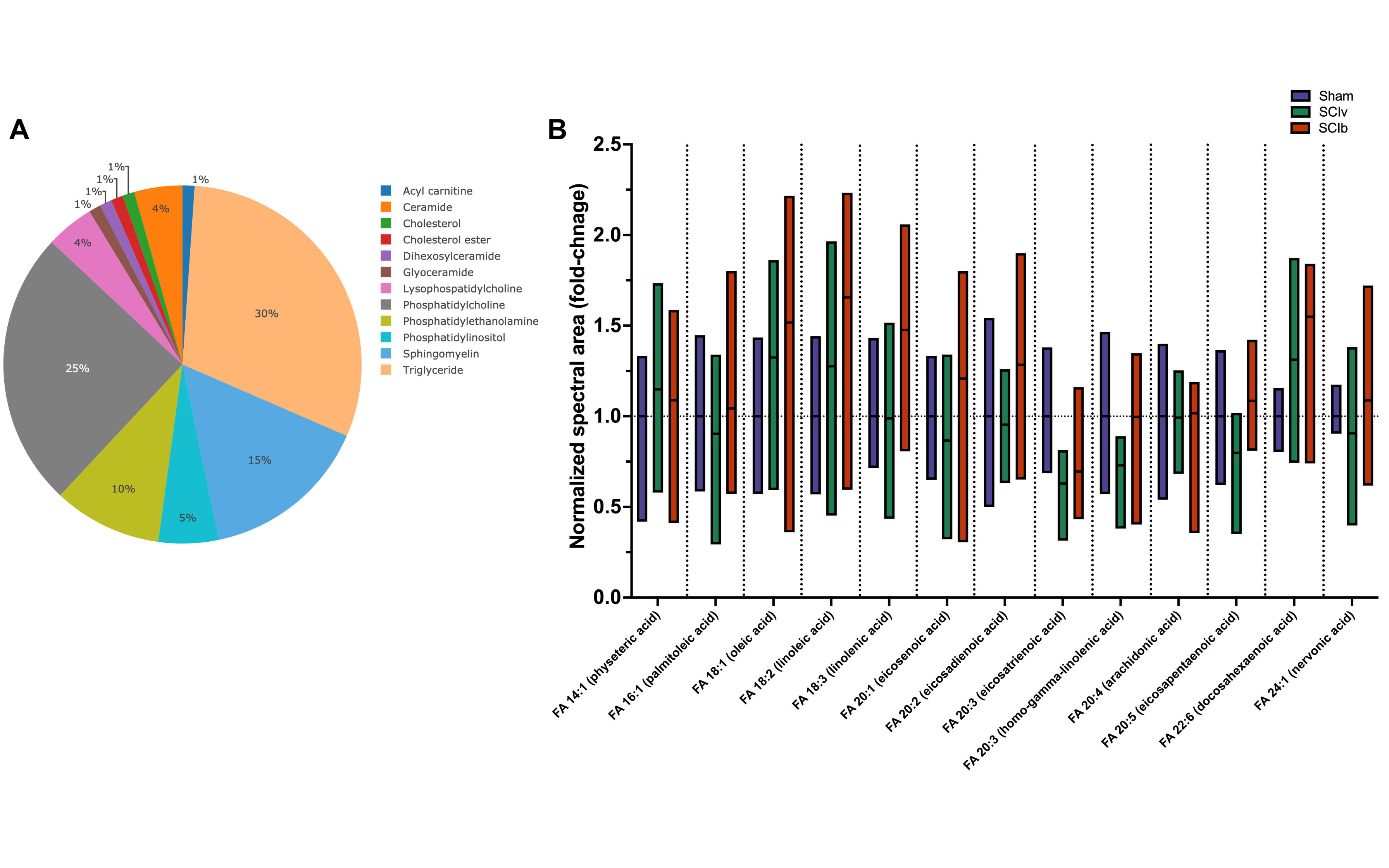

Supplement: Supplementary file 1 [file ijerph-20-06591-s001.zip › d7_serum_lipid_fig_s1.png]
